# Senolytics and Senomorphics Targeting p38MAPK/NF-κB Pathway Protect Endothelial Cells from Oxidative Stress-Mediated Premature Senescence

**DOI:** 10.3390/cells13151292

**Published:** 2024-07-31

**Authors:** Jingyuan Ya, Ulvi Bayraktutan

**Affiliations:** Academic Stroke, Mental Health and Clinical Neurosciences, School of Medicine, University of Nottingham, Nottingham NG7 2UH, UK

**Keywords:** endothelial cell, senescence, p38MAPK, NF-κB, blood–brain barrier, senotherapeutic, senolytic, senomorphic, aging, age

## Abstract

Oxidative stress is a prominent causal factor in the premature senescence of microvascular endothelial cells and the ensuing blood–brain barrier (BBB) dysfunction. Through the exposure of an in vitro model of human BBB, composed of brain microvascular endothelial cells (BMECs), astrocytes, and pericytes to H_2_O_2_, this study examined whether a specific targeting of the p38MAPK/NF-κB pathway and/or senescent cells could delay oxidative stress-mediated EC senescence and protect the BBB. Enlarged BMECs, displaying higher β-galactosidase activity, γH2AX staining, p16 expression, and impaired tubulogenic capacity, were regarded as senescent. The BBB established with senescent BMECs had reduced transendothelial electrical resistance and increased paracellular flux, which are markers of BBB integrity and function, respectively. Premature senescence disrupted plasma-membrane localization of the tight junction protein, zonula occludens-1, and elevated basement membrane-degrading matrix metalloproteinase-2 activity and pro-inflammatory cytokine release. Inhibition of p38MAPK by BIRB796 and NF-κB by QNZ and the elimination of senescent cells by a combination of dasatinib and quercetin attenuated the effects of H_2_O_2_ on senescence markers; suppressed release of the pro-inflammatory cytokines interleukin-8, monocyte chemoattractant protein-1, and intercellular adhesion molecule-1; restored tight junctional unity; and improved BBB function. In conclusion, therapeutic approaches that mitigate p38MAPK/NF-κB activity and senescent cell accumulation in the cerebrovasculature may successfully protect BBB from oxidative stress-induced BBB dysfunction.

## 1. Introduction

The accumulation of senescent cells in vivo constitutes one of the main causes of organismal aging and age-related diseases (ARDs). The widespread distribution of senescent cells in a variety of organs and lesions, such as atherosclerotic plaques, in aged versus young and healthy subjects provides evidence for this notion and pinpoints senescent cells as a potential therapeutic target [1,2,3]. Senescent cells lose their ability to self-renew and fail to conduct their normal function [4]. In addition, they secrete a cocktail of cytokines, chemokines, growth factors, and matrix metalloproteinases (MMPs), collectively known as SASP or the senescence-associated secretory phenotype. SASP spreads senescence to the neighboring cells (paracrine effect) and renders the microenvironment amenable to aging and ARD development [4]. Hence, the early detection and elimination of senescent cells and SASP may be of huge therapeutic importance in the delay or prevention of ARDs [5].

The blood–brain barrier (BBB) is a highly specific semi-permeable barrier between the blood and the brain parenchyma. It regulates the selective passage of solutes, nutrients, and chemicals from the blood to the brain and vice versa. Brain microvascular endothelial cells (BMECs) make up the most important cellular constituents of the BBB. They cover the entire inner surface of cerebral microvasculature and tightly control the paracellular flux through tight junctions that form between adjacent cells and are composed of several transmembrane and peripheral membrane proteins, including occludin, claudins, and zonula occludens [6].

Although chronological aging adversely affects the physiological characteristics of the cerebral endothelium and the BBB, these structural and functional changes are augmented in a variety of neurodegenerative conditions, notably vascular dementia, Alzheimer’s disease, and stroke [7,8,9]. Here, the increased availability of senescent BMECs in the microvasculature may promote a pro-inflammatory microenvironment and plays a key role in age-related BBB dysfunction [10]. A diverse range of stimuli, such as replication stress, mitochondrial dysfunction, genotoxic stress, oxidative stress, and oncogenic activation, can trigger cellular senescence through the activation of one of the two mechanisms, namely telomere shortening due to replicative exhaustion and DNA damage followed by the DNA damage response (DDR) due exposure to different acute sub-lethal stresses, notably oxidative stress [11,12]. Oxidative stress stems from an imbalance between the synthesis and metabolism of reactive oxygen species (ROS), such as superoxide anion and hydrogen peroxide (H_2_O_2_). ROSs accelerate telomere shortening, oxidase DNA bases, breakdown DNA strands, and as a result, potentiate DDR activity [13]. These, in turn, activate various mechanisms that lead to cellular senescence via the activation of cell-cycle checkpoints and mitochondrial dysfunction, which then produce more ROSs and help maintain this vicious circle in an active state [14].

**Figure 1:**
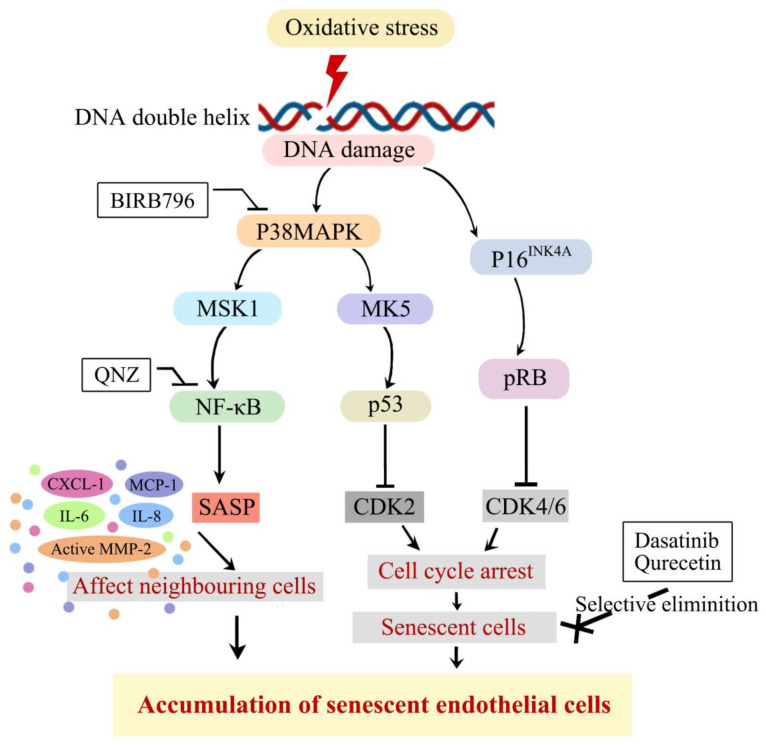
A schematic representation of mechanisms involved in oxidative stress-mediated induction of cellular senescence. Excessive bioavailability of oxidative stress promotes DNA damage and activates p38MAPK/p53 and p16/RB pathways which, in turn, inhibit the CDK2 and CDK4/6 enzymatic activities and induce cell-cycle arrest. Activation of p38MAPK/NF-κB pathway also promotes senescence through release of SASP. Therapeutic strategies targeting p38MAPK/NF-κB pathway or senescent cells themselves effectively attenuate accumulation of senescent cells. P38MAPK, p38 mitogen-activated protein kinase; MSK1, mitogen- and stress-activated protein kinase-1; NF-κB, nuclear factor kappa B; SASP, senescence-associated secretory phenotype; MK5, map kinase-activated protein kinase 5; CDK, cyclin-dependent kinase; pRB, phosphorylate retinoblastoma protein.

The current evidence indicates that p38MAPK/NF-κB is a key signaling pathway in the context of senescence. As illustrated in Figure 1, p38MAPK/NF-κB is activated in response to enhanced oxidative stress, DDR, and pro-senescence stimuli [15,16]. Once activated, p38MAPK elevates the release of several SASP components, e.g., IL-6, Il-8, and IL-1β, and, as a consequence, accelerates senescence in different cell lines, such as fibroblasts [17]. Similarly, the transcription factor NF-κB regulates the expression of genes that mediate the release of several pro-inflammatory factors, such as IL-6, IL-8, and granulocyte macrophage colony-stimulating factor (GM-CSF), major factors of SASP [4]. Given the prominent role of the p38MAPK/NF-κB pathway in a variety of major cellular events including proliferation, cell-cycle progression, and apoptosis [18], the present study aimed to investigate whether specific targeting of the components of this particular pathway may be of great benefit to protect against oxidative stress-induced senescence and ensure BBB function.

## 2. Materials and Methods

### 2.1. Cell Culture and Treatments

Human BMECs (HBMECs), human pericytes (HP), and human astrocytes (HA) were purchased from Neuromics (Minneapolis, MN, USA). All cells were cultured in their respective media (Sciencell Research Laboratories, San Diego, CA, USA) in a humidified atmosphere of 75% N_2_, 20% O_2,_ and 5% CO_2_ at 37 °C. Throughout the study, HBMECs (passages 6–8) were exposed to different experimental conditions. To determine the optimal condition that yields high levels of premature senescence without compromising overall viability, HBMECs were cultured in a complete medium containing different concentrations of H_2_O_2_ (100, 400, and 1000 μM) for 24, 48, or 72 h. A further 1, 6, or 12 days of incubation under normal experimental conditions followed these treatments.

BIRB796 or QNZ were added to the culture media of HBMECs being subjected to H_2_O_2_ to inhibit p38MAPK and NF-κB, respectively. In an additional treatment group, as the development of senescence took about 12 days, a cocktail of dasatinib (a tyrosine kinase inhibitor and a senolytic) and quercetin (a flavoprotein and a senomorphic) was added to HBMECs on day 10 of the culture for 24 h to eliminate senescent cells. The cells were then returned to normal culture conditions for 24 h before commencing the relevant experiments.

### 2.2. Assessment of Senescence-Associated β-Galactosidase Activity

HBMECs were seeded in 12-well plates and cultured to around 70% confluence in a specific culture medium. The SA-β-galactosidase (SA-β-gal) activity was evaluated using a beta-galactosidase staining kit (Ab102534, Abcam, Cambridge, UK) according to the manufacturer’s instructions. In brief, the cells were washed with warm PBS, fixed for 10 min with the fixation solution at room temperature, and incubated overnight in a staining solution at 37 °C. Cells with blue dye under a light microscope were regarded as senescent. To quantify the differences in β-galactosidase activity amongst different study groups, cells were photographed with a light microscope (DFC3000 G, Leica Microsystems, Wetzlar, Germany) under 10× magnification. Minimally, a total of 100 cells was counted manually in at least four randomly chosen areas of each slide before working out the percentage of SA-β-gal-positive cells. The average of four randomly chosen areas was used for statistical analyses. Each experiment was repeated at least three times using three different biological replicates.

### 2.3. LDH Cytotoxicity Assay

The CyQUANT LDH cytotoxicity Assay Kit (C20300, Invitrogen, Carlsbad, CA, USA) was used as per the manufacturer’s instructions to quantify the level of cellular cytotoxicity. Briefly, HBMECs (5 × 10^3^) were seeded in triplicate in 96-well plates and incubated overnight. On the following day, the media were replaced with 100 μL of fresh media containing 10 μL sterile water (spontaneous control), 10 μL 10× lysis buffer (maximum LDH activity control), and 10 μL H_2_O_2_ from different stocks to obtain a final concentration of 100, 400, 800, and 1600 μM. The culture plate was then incubated for 45 min before collecting 50 μL of sample media from each well and mixing it with the reaction mixture at a ratio of 1:1 for 30 min. The reactions were then terminated by the addition of the stop solution. The absorbances were then read at 490 nm and 680 nm, and the level of cytotoxicity was calculated using the following formulae. %Cytotoxicity = [(Compound-treated LDH activity − Spontaneous LDH activity)/(Maximum LDH activity − Spontaneous LDH activity)] × 100.

### 2.4. Establishment of an In Vitro Model of Human BBB

A triple cell-culture model of human BBB composed of HBMECs, HA, and HP was established as described before [19]. In brief, approximately 1 × 10^5^ HAs were seeded on the basolateral side of the transwell insert (polyester membrane, 12 mm diameter, 0.4 μm pore size, Corning Costar, Grays, UK). Once the cells adhered to the membrane, the inserts were inverted the right way and cultured in sterile 12-well plates to ~90% confluence. HBMECs (5 × 10^4^) subjected to different experimental conditions were then seeded onto the apical side of the inserts and cultured to full confluence. The inserts containing fully confluent HBMECs and HAs were transferred to fresh 12-well plates containing confluent HPs, to set the triple culture model of human BBB. BBB integrity and function were then evaluated by measurements of the transendothelial electrical resistance (TEER) and paracellular flux of sodium fluorescein (NaF, 50 μg/mL, 376D).

### 2.5. Tubulogenesis Assay

The level of tube-formation capacity on the growth-factor-reduced Matrigel (Corning, New York, NY, USA) was studied to evaluate the angiogenic capacity of the HBMECs. For this, the Matrigel was thawed at 4 °C overnight and pipetted into 96-well plates (50 μL per well) using pre-chilled tips on a cooling core. The well plates were incubated at 37 °C for 1 h to solidify the Matrigel. HBMECs (8 × 10^3^ cell/150 μL culture medium) subjected to different experimental conditions were seeded in 96-well plates pre-coated with Matrigel and incubated at 37 °C for 4 h. The formation of the tubular structures was visualized and photographed using the Digipad connected to a light microscope (DFC3000 G, Leica Microsystems, Wetzlar, Germany). Using the Angiogenesis Analyzer plugin for ImageJ software (version 1.52k, NIH, Bethesda, MD, USA), the characteristics of the tubule networks, i.e., the total number and the total length of the segments, were analyzed.

### 2.6. Immunocytochemistry

HBMECs seeded on glass coverslips were subjected to different experimental conditions before fixing (in 4% paraformaldehyde for 15 min) and permeabilizing (in 0.1% Triton X-100 for 15 min) at room temperature. After blocking with 1% bovine serum albumin in PBST (0.1% Tween20 in PBS) for 30 min at room temperature, the cells were incubated overnight at 4 °C with primary antibodies, including ZO-1 (33-9100, Thermofisher, Waltham, MA, USA), occludin (71-1500, Thermofisher), claudin-5 (35-2500, Theromofisher), and phospho-Histone H2AX (Ser139, 9718, Cell Signaling Technology, Danvers, MA, USA). On the following day, the cells were washed and incubated with FITC-labeled or Texas Red-labeled secondary antibodies (ab6785, an6717, ab6719, Abcam, Cambridge, UK) for 1 h at room temperature in the dark. The nuclei were stained with DAPI (4,6-diamidino-2-phenylindole). The coverslips were then mounted onto glass slides using a mounting medium (Vector Laboratories, Peterborough, UK). The cells were visualized by fluorescence microscopy (DFC3000 G, Leica Microsystems, Wetzlar, Germany). The nuclei with red-stained foci were considered as γH2AX-positive cells. The total cell numbers were determined by counting all DAPI-stained nuclei. A minimum of 40 nuclei were counted manually in at least four randomly chosen areas of each slide before working out the percentage of γH2AX-positive cells. The average of four randomly chosen areas was used for statistical analyses. Each experiment was repeated at least three times using three different biological replicates.

### 2.7. Protein Extraction and Quantification

After exposure to different experimental conditions, the cells were washed twice with ice-cold PBS and then scraped in lysis buffer (1× RIPA buffer, 10 μL/mL protease inhibitor cocktail, 1 mM sodium orthovanadate). The supernatant was collected from the cell lysate after centrifuging at 14,000× *g* (4 °C) for 15 min. Protein concentrations were detected using the BCA protein assay kit (23227, Thermofisher, Waltham, MA, USA) as per the manufacturer’s instructions.

### 2.8. Western Blotting

Protein samples (20–100 μg) were mixed with 4× LDS (4% lithium dodecyl sulfate) sample buffer (MPSB, Sigma, Burlington, MA, USA) in a ratio of 3:1 and heated at 75 °C for 5 min to open the tertiary/quaternary structures by denaturation. After separation on 15% SDS-polyacrylamide gel (8% for ZO-1), the protein samples were transferred to a PVDF membrane. The membrane was blocked with 5% bovine serum albumin (BSA) at room temperature for 1 h before incubating overnight with a primary antibody at 4 °C. To this end, ZO-1 (33-9100), occludin (71-1500), and claudin-5 (35-2500) antibodies from Theromofisher, phospho-p38MAPK (Thr180/Tyr182, 9216), p38MAPK (8690), phospho-NF-κB p65 (Ser536, 13346), NF-κB (8242) antibodies from Cell Signaling Technology, and p16 (ab51243) and anti-beta actin (ab8226) antibodies from Abcam were used. The membranes were then incubated with IRDye-labeled (800CW/680CW) secondary antibodies. The protein bands were detected using the Odyssey Fc System before analyzing their intensities via Image Studio software (5.0, Li-cor Biotechnology, Lincoln, NE, USA).

### 2.9. Telomere Length Measurement

Genomic DNA from HBMECs was extracted with the DNeasy Blood & Tissue Kit (69504, Qiagen, Germantown, MD, USA). The telomere length of the genomic DNA samples was measured by real-time PCR using the Absolute Human Telomere Length Quantification qPCR Assay Kit (ScienCell Research Laboratories, Carlsbad, CA, USA). The average telomere length on each chromosome end was calculated as per the manufacturer’s instructions using the formulae based on the Cq value acquired by the Agilent Stratagene MX3000P Quantitative RT-PCR System (Santa Clara, CA, USA).

### 2.10. Gelatin Zymography

After exposure to different experimental conditions, HBMECs were washed with warm PBS and incubated in serum-free DMEM for 20 h before collecting and centrifuging the supernatant at 300× *g* for 10 min to remove the cells and debris. Twenty μL of supernatant were mixed with 4× LDS Sample Buffer in a ratio of 1:3 and run on 10% SDS-polyacrylamide gel containing 0.1% (*w*/*v*) gelatin for 3 h at 100V in the cold room. The gel was washed in 2.5% Triton X-100 twice, first for 30 min and then for 1 h at room temperature before incubating for 16 h at 37 °C in the incubation buffer (50 mM Tris-HCl pH7.5, 10 mM CaCl_2_, 1 μM ZnCl_2_, 200 mM NaCl). The gels were then stained with staining solution (0.1% [*w*/*v*] Coomassie blue dye, 40% deionized water, 50% methanol, and 10% acetic acid) for 1 h and de-stained 4 times for 10 min each time using de-staining solution (50% deionized water, 40% methanol, and 10% acetic acid). MMP activity was detected as clear bands against a dark blue background. The gels were scanned and analyzed using the Odyssey Fc System (Li-cor Biotechnology, Lincoln, NE, USA).

### 2.11. Proteome Profiler Cytokine Array

After exposure to different experimental conditions, BMECs were washed twice with PBS and cultured with serum-free and growth-factor-free media for 24 h. Cell-culture media were collected and centrifuged at 250× *g* for 5 min to remove the debris. The supernatant was then condensed 8 times by centrifugation at 4000× *g* for 20 min using a Centrifugal Filter (Merck, Taufkirchen, Germany). A Proteome Profiler Human Cytokine Array Kit (R&D Systems) was used as per the manufacturer’s instructions to detect cytokines. Briefly, the samples were mixed with the detection-antibody cocktail, then incubated with the blocked membrane spotted with the antibodies at 4℃ overnight. After washing, the membranes were incubated with Streptavidin-HRP solution and activated by Chemi Reagent Mix. The chemiluminescent signal of the dots on the membrane was detected using the Odyssey Fc system, and the blotting density was measured by ImageJ software (5.0, National Institutes of Health, Bethesda, MD, USA).

### 2.12. Statistical Analysis

The data are displayed as the mean value ± standard error of the mean (SEM) from a minimum of three independent experiments. Differences among groups were determined using an unpaired *t*-test. *p* < 0.05 was considered as significant.

## 3. Results

### 3.1. H_2_O_2_ Induced Senescence in HBMECs in a Dose- and Time-Dependent Fashion

**Figure 2:**
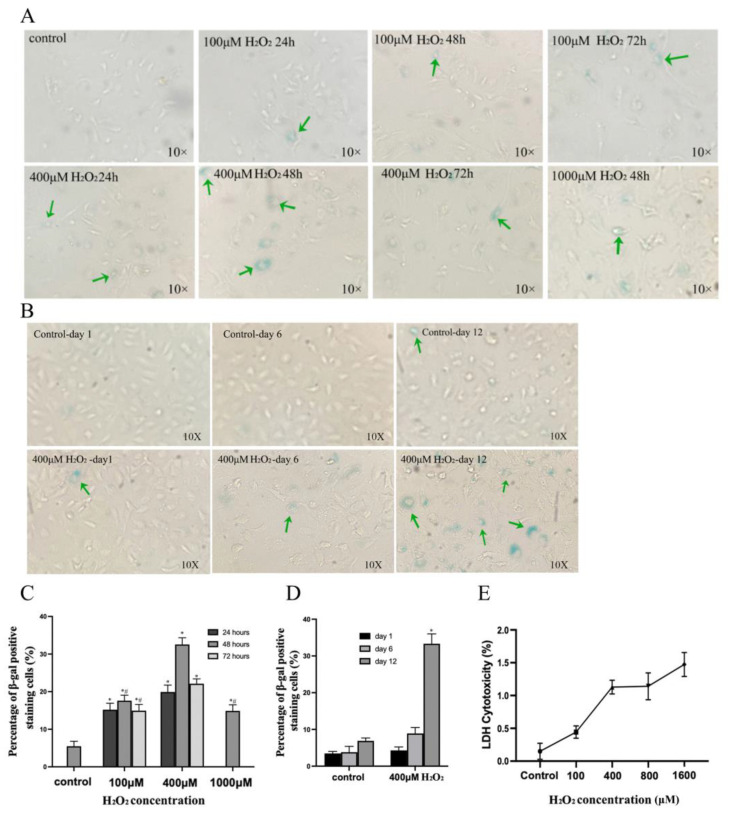
Time- and concentration-dependent effects of H_2_O_2_ on HBMECs. (**A**) Senescent cells emerged in cells treated with increasing concentrations of H_2_O_2_ for 24–72 h. (**B**) Twelve days appeared to be the best time period for the development of senescence following exposure to 400 μM of H_2_O_2_. (**C**,**D**) Percentage of cells reaching senescence, as ascertained by SA-β-gal positive staining, in response to different concentrations of H_2_O_2_ and post-H_2_O_2_ incubation period. (**E**) Level of LDH cytotoxicity in response to different concentrations of H_2_O_2_. The green arrows indicate some of the SA-β-gal positive cells in different experimental groups. * indicates statistically significant differences compared to control group. ^#^ indicates statistically significant differences between 100 and 1000 μM treatment groups and corresponding 400 μM group. Data are expressed as mean ± SEM from three independent experiments.

Exposure to incremental concentrations_._ (100, 400 and 1000 μM) of H_2_O_2_ for increasing periods of time (24, 48, and 72 h) to induce oxidative stress resulted in a higher prevalence of SA-β-gal positive cells, a marker of senescence, in cell populations subjected to 400 μM of H_2_O_2_ for 48 h before a further culture in normal conditions for 12 days. The LDH cytotoxicity assay observed an increase of 1.13% cytotoxicity with 400 μM H_2_O_2_ (Figure 2A–E).

### 3.2. H_2_O_2_ Induced Morphological Changes and Nuclear Damage in Senescent Cells

**Figure 3:**
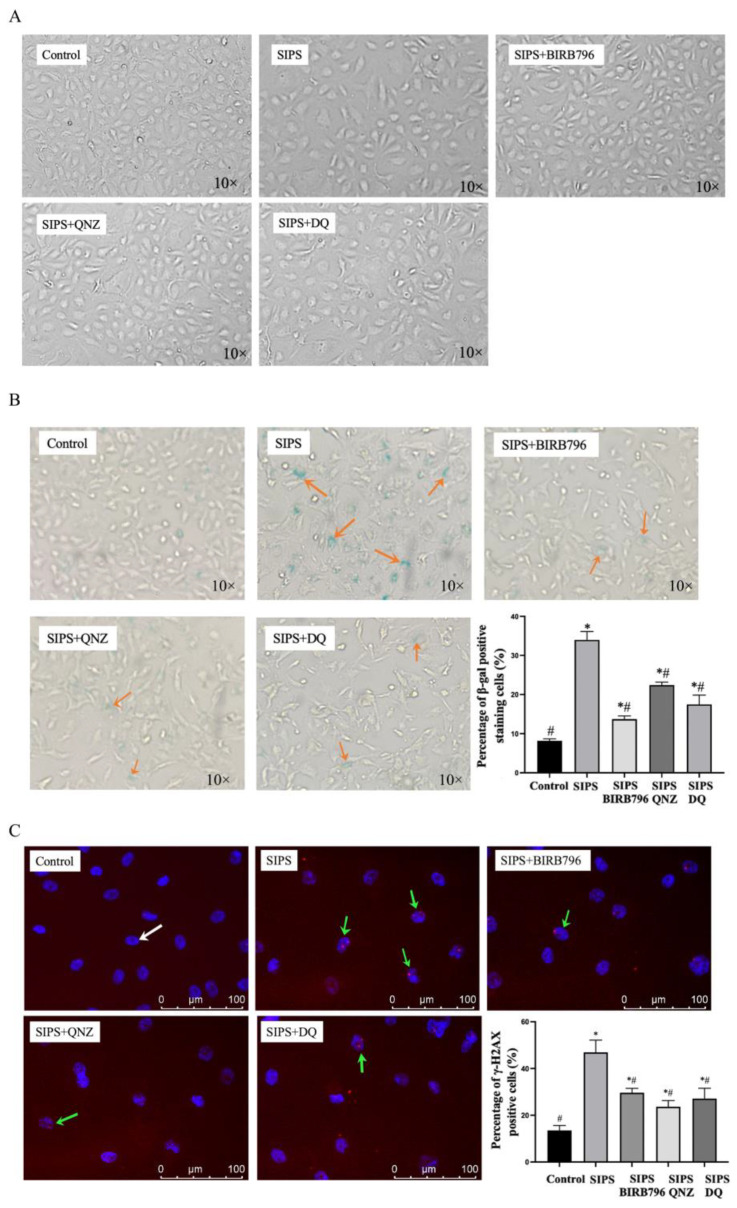
Exposure to H_2_O_2_ led to enlarged cellular morphology (**A**) and elevated the number of cells stained positive for SA-β-gal (**B**, orange arrow) and γH2AX (**C**, green arrow) where normal nuclear staining with DAPI is illustrated by a white arrow. * *p* < 0.05 compared to the control group, ^#^ *p *< 0.05 compared to the SIPS group.

Exposure to H_2_O_2_ led to enlarged cell morphology and increases in SA-β-gal activity and γH2AX staining, a DNA double-strand break marker, in HBMECs 12 days after the cessation of exposure. Co-treatment of cells with an inhibitor of p38MAPK (BIRB796) or NF-κB (QNZ) or the combination of dasatinib and quercetin (D+Q) attenuated the impact of H_2_O_2_ on all these elements (Figure 3A–C).

### 3.3. H_2_O_2_ Evoked an Increase in p16 Expression

**Figure 4:**
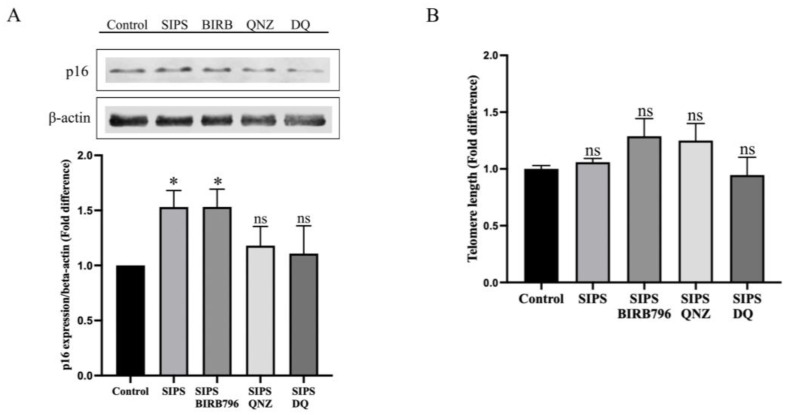
Effect of treatments with H_2_O_2_ on p16 expression and telomere length. A significant increase in the expression of cyclin-dependent kinase inhibitor p16 was detected by Western analyses in prematurely senescent cells that were selectively suppressed by treatments with an inhibitor of NF-κB (QNZ) and D+Q (**A**). No significant difference was observed in telomere length in any of the experimental groups in the absence or presence of inhibitors (**B**). Data are presented as mean ± SEM from three independent experiments. * *p* < 0.05 compared to the control group, ns = not significant compared to control and/or SIPS group.

Exposure of HBMECs to H_2_O_2_ evoked significant increases in the expression of cyclin-dependent kinase inhibitor p16, which was selectively suppressed by treatments with an inhibitor of NF-κB (QNZ) and D+Q but not treatment with a p38MAPK inhibitor, BIRB-796 (Figure 4A). However, no significant difference was observed in telomere length between the control cells and those subjected to H_2_O_2_ in the absence or presence of BIRB-796, QNZ, or D+Q, implying that oxidative stress-mediated SIPS may be developed by DDR through a telomere-independent mechanism (Figure 4B).

### 3.4. Impact of H_2_O_2_ on p38MAPK and NF-κB Phosphorylation

**Figure 5:**
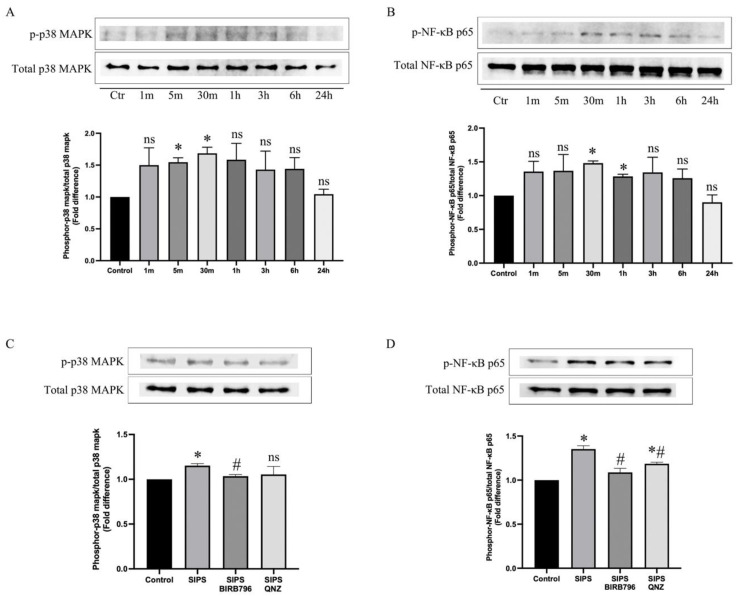
Representative images showing the impact of H_2_O_2_ on p38MAPK and NF-κB phosphorylation. H_2_O_2_ evoked p38MAPK (**A**) and NF-κB (**B**) phosphorylation in a time-dependent manner. The increases observed in both p38MAPK and NF-κB activity within 30 min of exposure to H_2_O_2_ were significantly suppressed by BIRB796, a p38MAPK inhibitor where the impact of QNZ was specific to NF-κB (**C**,**D**). Data are presented as mean ± SEM from 3 independent experiments. * *p *< 0.05 compared to the control group, ^#^ *p *< 0.05 compared to the SIPS group, ns = not significant compared to control and/or SIPS group.

To determine whether exposure to oxidative stress may phosphorylate p38MAPK and/or NF-κB in HBMECs in a time-dependent manner, the cells were treated with 400 μM of H_2_O_2_ for up to 24 h. These led to significant increases in p38MAPK and NF-κB phosphorylations within 5 min and 30 min, respectively (Figure 5A,B).

While BIRB796 suppressed the activation of both p38MAPK and NF-κB in cells subjected to H_2_O_2_ for 30 min, treatment with QNZ only suppressed the activation of NF-κB without affecting that of p38MAPK, indicating that NF-κB acts as a downstream effector to p38MAPK (Figure 5C,D and Figures S1–S3).

### 3.5. Premature Senescent HBMECs Fail to Form a Functional BBB

**Figure 6:**
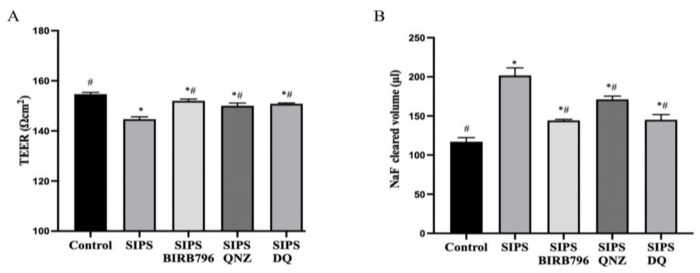
HBMECs senescence adversely affect the characteristics of blood–brain barrier. An in vitro model of human BBB established with human astrocytes, pericytes, and senescent HBMECs led to significantly decreased transendothelial electrical resistance (TEER) (**A**) and increased paracellular flux (**B**) of a low molecular weight permeability marker, sodium fluorescein (NaF). Treatments with BIRB796 (a p38MAPK inhibitor), QNZ (an NF-κB inhibitor), and a combination of dasatinib and quercetin (DQ) markedly reduced the impact of SIPS on both parameters. Data are expressed as mean ± SEM from three independent experiments. * *p* < 0.05 compared to the control group, ^#^ *p* < 0.05 compared to the SIPS group.

Similar to replicative senescent ECs [20,21], prematurely senesced HBMECs also failed to form fully functional barriers as ascertained by decreases in TEER and increases in the paracellular flux of a low molecular weight paracellular flux marker, NaF. The selective elimination of senescent ECs by D+Q and inhibition of p38MAPK and NF-κB significantly attenuated the barrier-disruptive effect of SIPS but failed to completely neutralize the changes observed in barrier integrity and function (Figure 6A,B).

### 3.6. Premature Senescent HBMECs Lack Angiogenic Capacity

**Figure 7:**
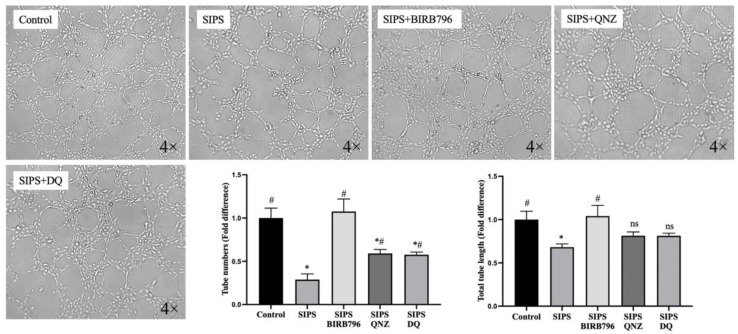
Senescence diminishes the angiogenic capacity of HBMECs. Cells subjected to H_2_O_2_ formed significantly fewer and shorter tubules on Matrigel compared to those cultured under normal conditions. Treatment with BIRB796, a p38MAPK inhibitor, completely restored the angiogenic capacity while treatments with QNZ, an NF-κB inhibitor, and a cocktail of dasatinib and quercetin (DQ) were not as effective despite attenuating the impact of H_2_O_2._ Data are expressed as mean ± SEM from three independent experiments. * *p *< 0.05 compared to the control group, ^#^ *p* < 0.05 compared to the SIPS group, ns = not significant compared to control and/or SIPS group.

A tubulogenic assay measuring the ability of endothelial cells to form tubules on Matrigel showed that SIPS adversely affected the capacity of HBMECs to form capillary-like structures. Although the inhibition of both p38MAPK and NF-κB, as well as the elimination of senescent cells by D+Q, negated the SIPS-mediated decreases in the number and length of the tubules observed, the magnitude of improvements was much better with BIRB796, a p38MAPK inhibitor (Figure 7).

### 3.7. SIPS Distinctly Affects Tight Junction Protein Expression and Localisation and Activates Matrix Metalloproteinase-2

**Figure 8:**
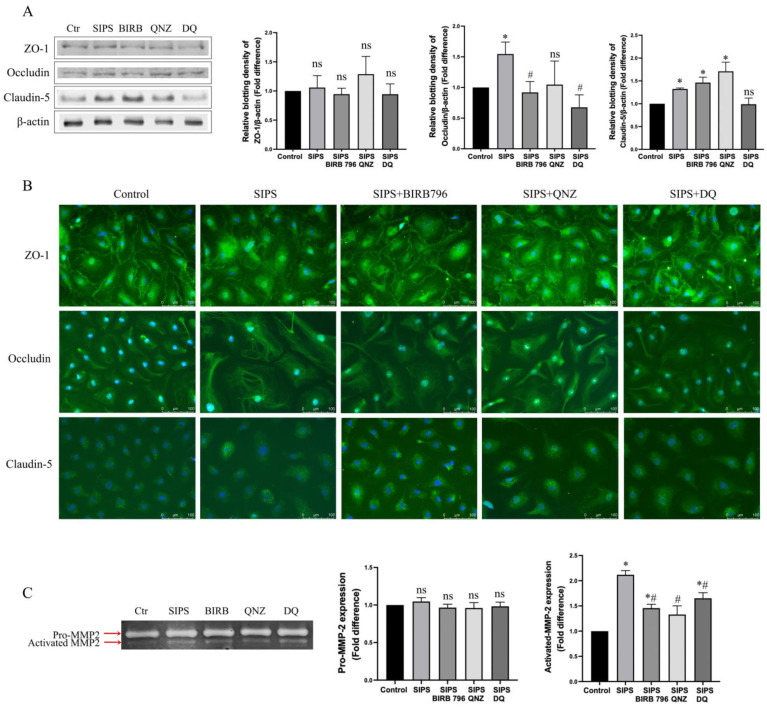
Stress-induced premature senescence affected the subcellular distribution of tight junction proteins. Compared to controls, H_2_O_2_ increased total expression of occludin and claudin-5 without affecting that of ZO-1 (**A**). Treatment with H_2_O_2_ perturbed plasma-membrane localization of zonula occludens-1 (ZO-1) without affecting those of occludin and claudin-5. Co-treatment of cells with H_2_O_2_ and an inhibitor for p38MAPK (BIRB796), NF-κB (QNZ), or a combination of dasatinib and quercetin (DQ) restored the plasma-membrane expression of ZO-1 (**B**). While levels of pro-MMP-2 remained unaffected in all experimental groups, a significantly increased level of activated MMP-2 was observed in SIPS group only. Treatments with BIRB796, QNZ, and DQ suppressed the oxidative stress-evoked MMP-2 activation (**C**). Due to high molecular weight of ZO-1, the same protein samples used for the detection of occludin, claudin-5, and β-actin were run on separate gels. As cell-culture media rather than cell lysates were used for gelatin zymography studies, no housekeeping protein could be used as loading control [22,23]. Data are expressed as mean ± SEM from three independent experiments. * *p *< 0.05 compared to the control group, ^#^ *p *< 0.05 compared to the SIPS group, ns = not significant compared to control and/or SIPS group.

Investigation of the subcellular mechanisms involved in SIPS-induced BBB disruption showed that, while SIPS did not alter ZO-1 protein expression, it elevated those of occludin and claudin-5. Interestingly, albeit insignificant compared to the SIPS group, co-treatments with BIRB796 and QNZ caused further increases in HBMEC claudin-5 levels. In SIPS HBMECs, ZO-1 showed disruptive plasma-membrane staining and appeared to localize to the cytoplasm and perinuclear area. Treatments with BIRB796, QNZ, and D+Q restored plasma-membrane localization of ZO-1 in HBMECs. In SIPS HBMECs, occludin appeared in the cytosol in a somewhat tubular fashion, which was not greatly affected by any of the treatment regimens. In contrast, claudin-5 congregated largely in the perinuclear region of cells exposed to SIPS in the absence or presence of the abovementioned senotherapeutics (Figure 8A,B).

To investigate whether the breakdown of the extracellular matrix may contribute to SIPS-induced BBB breakdown, the levels of pro- and active MMP-2 and -9 were examined in the control versus the SIPS HBMECs by gelatin zymography. In this study, only pro-MMP-2 and active MMP-2 could be detected. While pro-MMP-2 levels remained the same in all the experimental groups, SIPS significantly raised the level of active MMP-2. Treatments with p38MAPK/NF-kB pathway inhibitors and D+Q markedly reduced these increases without normalizing the levels of pro-MMP2 (Figure 8C).

### 3.8. Oxidative Stress Promotes the Expression of Senescence-Associated Secretory Phenotype

**Figure 9:**
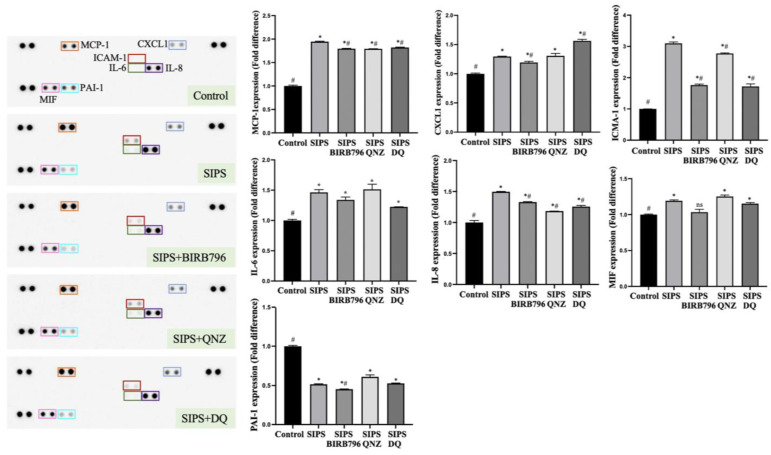
Senescence-induced adoption of a pro-inflammatory phenotype in human brain microvascular endothelial cells. Cells exposed to H_2_O_2_ secreted significantly higher levels of MCP-1, CXCL-1, ICAM-1, IL-6, IL-8, and MIF-1 and lower levels of PAI-1. Inhibition of p38MAPK (by BIRB796) and NF-κB (by QNZ) and treatment with a cocktail of dasatinib and quercetin (DQ) partially suppressed the overexpression of the MCP-1, ICAM-1, and IL-8. Treatment with BIRB796 also suppressed the expression of CXCL-1. The expression of cytokines was quantified using ImageJ based on the densitometric analysis of the dot blot duplicates. * *p *< 0.05 compared to the control group, ^#^ *p *< 0.05 compared to the SIPS group, ns = not significant compared to control and/or SIPS group.

HBMECs exposed to H_2_O_2_ acquired SASP, as evidenced by increases in the expression of pro-inflammatory elements, monocyte chemoattractant protein-1 (MCP-1), CXC motif chemokine ligand 1 (CXCL1), intercellular adhesion molecule-1 (ICAM-1), interleukin-6 (IL-6), IL-8 and macrophage migration inhibitory factor (MIF), and a decrease in plasminogen activator inhibitor-1 (PAI-1) level compared to control cells (Figure 9).

The selective clearance of senescent cells by D+Q and the inhibition of p38MAPK and NF-κB diminished SIPS-promoted secretion of MCP-1, ICAM-1, and IL-8. The impact of SIPS on CXCL1 expression was specifically suppressed by BIRB796. Treatment with D+Q caused further elevations in CXCL1 levels (Figure 9). None of the therapeutic options had markedly influenced the effect of SIPS on IL-6 or MIF expressions. In contrast, BIRB796 caused a further decrease in PAI-1 levels compared to control cells and those subjected to H_2_O_2_ alone.

## 4. Discussion

The accumulation of senescent cells in vivo may contribute to the pathogenesis of various ARDs. Specifically, accumulation of BMECs in cerebral vasculature is likely to evoke structural and functional damage to BBB and may contribute to the pathogenesis of several neurovascular conditions associated with the aging process, including stroke [24,25]. BBB breakdown, evidenced by increased permeability to radiocontrast agents, is also a common occurrence during the process of physiological aging [26]. BMECs play a key role in the establishment and maintenance of BBB in that they not only restrict paracellular flux through the formation of tight junctions that seal the gaps between adjacent cells but also limit transcellular transport. Moreover, BMECs help adjust the cerebral flow, control the transmigration of immunocytes in the central nervous system, and secrete a wide range of active compounds, e.g., nitric oxide, endothelin-1, cytokines, chemokines, and enzymes that regulate immune responses, vascular tone, coagulation, angiogenesis, and neurogenesis [27,28].

To corroborate whether the presence of senescent HBMECs prompts significant impairment in BBB integrity and function, a triple culture model of human BBB composed of astrocytes, pericytes, and young or senescent HBMECs was employed in this study. To mimic oxidative stress-induced senescence, cells were subjected to H_2_O_2_ (400 μM) for 48 h, and those cells displaying enlarged morphology and nuclear damage (ascertained by γH2AX staining), as well as higher SA-β-gal activity, were considered as senescent. Since the prevalence of cells possessing these characteristics was considerably higher in cells kept in normal culture conditions for 12 days after exposure to H_2_O_2_, these particular cells were used to set up the BBB, which revealed marked decreases in TEER values and increases in the paracellular flux compared to the BBB model set up using young HBMECs. These indicated that, through their physical presence and/or the compounds they release, senescent ECs evoke significant impairments in both the integrity and function of the BBB, which in turn propose these cells as potential therapeutic targets to maintain neurovascular homeostasis.

Previous studies focusing on the correlation between EC senescence and BBB characteristics attributed barrier hyperpermeability and dysfunction in both human umbilical vein EC (HUVEC) monolayers and a multicellular model of human BBB, containing replicative senescent BMECs, to changes in the distribution and availability of the tight junction proteins, including ZO-1, occludin and claudin-5 [20,21]. The co-culture of senescent HUVECs with non-senescent cells led to marked decreases in occludin and claudin-5 expressions between senescent cells and along the entire periphery of non-senescent cells lining senescent HUVECs [20,21]. Similarly, the co-culture of young HBMECs with replicative senescent outgrowth ECs, a functional subtype of endothelial progenitor cells capable of differentiating into mature endothelial cells, substantially diminished ZO-1 expression between adjacent cells and suppressed the tubulogenic capacity [29,30]. In our study, disrupted plasma-membrane staining and increased cytoplasm staining of ZO-1 were observed in prematurely senescent HBMECs, and treatments with BIRB796, QNZ, and D+Q prevented the re-distribution of ZO-1. The level of expression and subcellular localization of occludin and claudin-5 did not differ in cells treated with H_2_O_2_ in the absence or presence of the abovementioned inhibitors. Despite the observation of an initial increase of 1.13% cytotoxicity with 400 μM H_2_O_2_ in HBMECs, cellular viability in the long run appeared to be unaffected. Indeed, 12 days after exposure to H_2_O_2_, the cellular viability rates, assessed by a trypan blue exclusion test, remained similar between the H_2_O_2_ and control groups, being 96.19% vs. 95.52%, respectively. This implies that the differences observed in BBB integrity and function after exposure to H_2_O_2_ do not stem from cytotoxicity and ensuing reductions in cell numbers.

In addition to differences in tight junctional complex formation, the current study shows that an increase in MMP-2 activity may also be instrumental in senescence-mediated BBB breakdown. MMPs are a group of endopeptidases that are expressed as inactive zymogens and are subsequently activated by other extracellular proteases, hypoxia, or oxidative stress. Once activated, MMP-2 plays a key role in the regulation of blood vessel formation and remodeling, as well as tissue repair and regeneration [31]. However, MMP-2 can also compromise vascular integrity and trigger BBB leakage. We observed that senescent endothelial cells released higher levels of activated MMP-2 into the culture medium, which were neutralized by BIRB796, QNZ, and D+Q treatments. It is possible that the acquisition of SASP and the accompanying elevated release of a series of inflammatory mediators by senescent HBMECs, notably MCP-1, CXCL1, ICAM-1, IL-6, IL-8, and MIF, may also accentuate the barrier-disruptive effects of MMP-2 through the involvement of different mechanisms [22,23]. The overproduction of MCP-1, CXCL1, ICAM-1, IL-6, and IL-8 in senescent endothelial cells is also documented in previous studies, which suggests persistent DNA damage as the causal effect in the formation of SASP [32,33,34]. Once established, SASP reinforces the senescent state, spreads it to the neighboring cells, and participates in chronic inflammation, tissue remodeling, and immune modulation [35]. The observation of considerably higher levels of γH2AX staining in HBMECs cultured for 12 days after exposure to H_2_O_2_ compared to those kept in culture for shorter periods, i.e., 1 or 6 days, confirms the link between persistent DNA damage and the acquisition of SASP.

Migration, growth, and differentiation of ECs are required for the formation of new blood vessels, a.k.a. angiogenesis, which is a crucial element in neurovascular hemodynamic recovery after ischaemic cerebral tissue injury. To explore whether the angiogenic capacity of HBMECs declines with senescence, a tube-formation assay that measures the ability of ECs, plated on Matrigel at subconfluent densities to form capillary-like structures, was conducted. Decreases in the total number and total length of the tubules observed in the senescent HBMEC group prove that SIPS adversely affects various angiogenesis-related factors. These include ECs’ adhesive, proliferative, migratory, and angiogenic cytokine and proteolytic enzyme secretory capacity, which are required for overall tubulogenic activity [36].

Recent evidence suggests that environmental stresses, such as ROSs, can induce premature cellular senescence without critical telomere shortening [37]. The present study confirms this finding and reports an increased expression of p16, a cyclin-dependent kinase inhibitor, in HBMECs exposed to H_2_O_2_. Through its action on CDK4/6 kinases, p16 prevents the phosphorylation of retinoblastoma (Rb) family proteins and suppresses the release of E2F, a translation factor known to be crucial for cell-cycle progression. These, in turn, trigger G1 cell-cycle arrest and consequently promote senescence [38]. This study also reports that ROSs induce cell-cycle arrest and the transcription of senescence-associated genes through the activation of the p38MAPK/NF-κB signaling pathway components in a time-dependent fashion, implying both p38MAPK and NF-κB as potential targets to delay or attenuate SIPS. Given that senescence cannot be reversed by any of the current clinical interventions, this may be of significant (therapeutic) value. Due to its role in senescence growth arrest, in part by activation of the pRb/p16 pathway, p38MAPK had previously attracted some attention [21,39]. In the present study, the attenuation of senescence-related changes in SA-β-gal activity and γH2AX staining and the partial restoration of tubulogenesis and BBB characteristics with BIRB796 emphasize the importance of the p38MAPK signaling pathway in the management of premature senescence induced by environmental stresses. Suppression of p38MAPK may also prevent the spread of senescence to the neighboring cells through a paracrine mechanism involving NF-κB transcriptional activity [40]. Hence, treatments with QNZ rather expectedly reduced the impact of SIPS on SA-β-gal activity, γH2AX staining, and partially improved tubulogenesis and BBB integrity and function. By removing the burden of senescent cells on their microenvironment and remote tissue, senolytics are expected to improve neurovascular function. In support of this notion, treatment with D+Q diminished the SA-β-gal and γH2AX staining in prematurely senescent HBMECs and enhanced their tubulogenic capacity and ability to form a functional BBB. D+Q makes up an effective cocktail of senolytics with a significant anti-aging function [41]. Dasatinib is a small-molecule tyrosine kinase inhibitor that evokes apoptosis and abates the viability of senescent adipocytes [42]. Quercetin, on the other hand, is a flavoprotein with potent anti-oxidant and immunoprotective effects [43]. When used together, D and Q decrease the secretion of pro-inflammatory cytokines and major SASP factors in a more effective manner [44]. In accordance with these observations, treatment with D+Q markedly reduced the secretion of MCP-1, ICAM-1, and IL-8 in the current study. Albeit insignificant, a similar trend was also observed in the IL-6 levels. The inhibition of the p38MAPK/NF-κB pathway components displayed similar effects on these SASP elements, supporting the hypothesis that this pathway on its own may be sufficient for the development of SASP [45]. However, greater decreases in ICAM-1 and IL-8 levels with BIRB796 and the inefficacy of QNZ in subsiding CXCL1 expression suggest that additional downstream effectors, other than NF-κB, may also be involved in the p38MAPK-mediated appearance of SASP. It is of note that the pro-inflammatory cytokines IL-6, IL-8, MCP-1, and CXCL-1 stimulate the release of other inflammatory factors, trigger the recruitment and activation of immune cells, and accelerate cellular senescence in the vasculature that collectively promotes atherosclerotic disease development and plaque vulnerability [46,47,48]. Interestingly, despite reducing SIPS-induced increases in p16 expression, neither QNZ nor D+Q brought its levels closer to that seen in control cells. BIRB796 had no effect on p16 expression. In addition, none of the treatment regimens had any impact on telomere length. Taken together, these findings bestow a prominent role on SASP in oxidative stress-evoked EC premature senescence.

## 5. Conclusions

This study suggests that enhanced oxidative stress status during the aging process may contribute to the premature senescence of endothelial cells and, consequently, compromise the integrity and function of BBB. The inhibition of p38MAPK/NF-κB pathway components and the selective elimination of senescent cells from the vasculature may help protect the BBB from environmental damage promoted by oxidative stress and the ensuing inflammatory responses.

## Data Availability

Data are available on reasonable request from the authors.

## Supplementary Materials

The following supporting information can be downloaded at https://www.mdpi.com/article/10.3390/cells13151292/s1: Figure S1: Original Western blots for Figure 3. Figure S2: Original Western blots for Figure 4. Figure S3: Original Western blots and zymography images for Figure 7.

## Abbreviations

P38MAPK, p38 mitogen-activated protein kinase; NF-κB, nuclear factor kappa B; SIPS, stress-induced premature senescence; BBB, blood–brain barrier; HBMEC, human brain microvascular endothelial cell; HP, human pericyte; HA, human astrocyte; ARD, age-related disease; MMP, matrix metalloproteinase; SASP, senescence-associated secretory phenotype; DDR, DNA damage response; ROS, reactive oxygen species; IL, interleukin; TEER, transendothelial electrical resistance; DQ, dasatinib and quercetin; MCP-1, monocyte chemoattractant protein-1; CXCL1, CXC motif chemokine ligand 1; ICAM-1, intercellular adhesion molecule-1; MIF, macrophage migration inhibitory factor; and PAI-1, plasminogen activator inhibitor-1.
