# Supplementary material for: Senolytics and Senomorphics Targeting p38MAPK/NF-κB Pathway Protect Endothelial Cells from Oxidative Stress-Mediated Premature Senescence
Source: Cells. 2024 Jul 31;13(15):1292. doi: 10.3390/cells13151292 (PMC11311971; doi:10.3390/cells13151292)
Supplement: Supplementary file 1 [file cells-13-01292-s001.zip › cells-3069018-supplementary.pdf]

Supplementary materials

Figure S1. Original western blots for figure 3. Bands on the membranes were captured using two different channels simultaneously i.e. 800 nm (anti-rabbit secondary antibody) and 680 nm channels (anti-mouse secondary antibody).

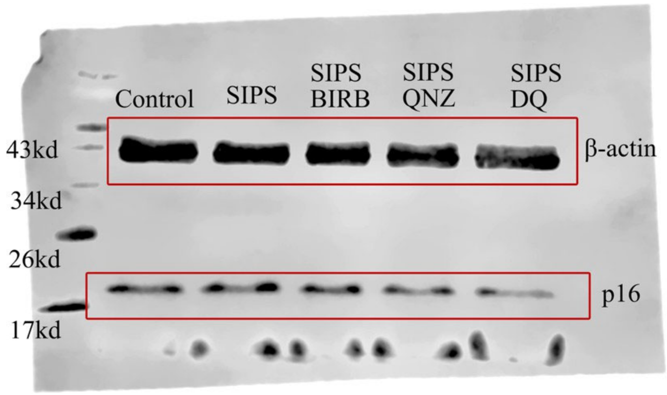

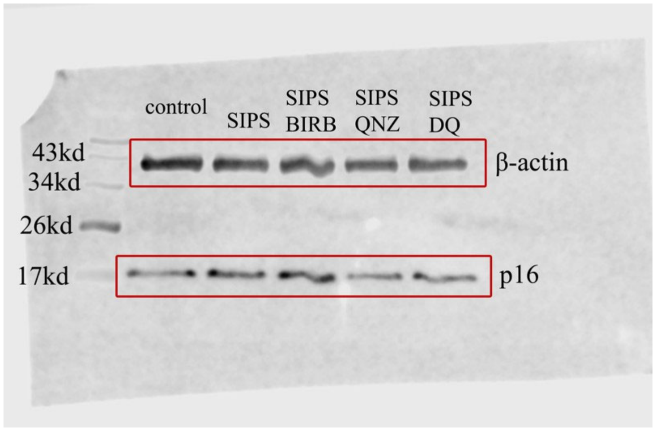

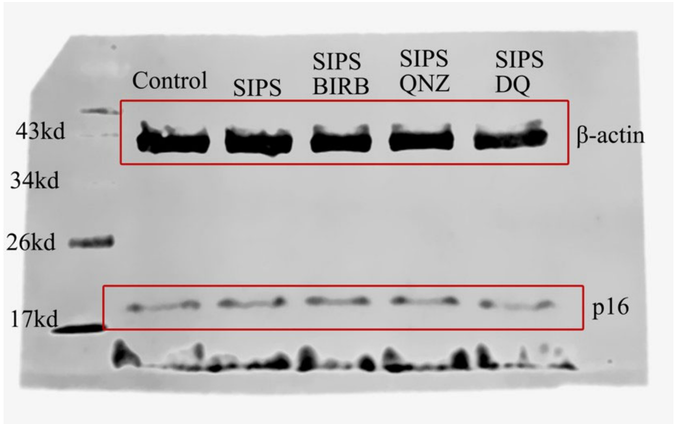

Figure S2. Original western blots for figure 4. Bands on the membranes were captured using two different channels simultaneously i.e. 800 nm (anti-rabbit secondary antibody) and 680 nm channels (anti-mouse secondary antibody).

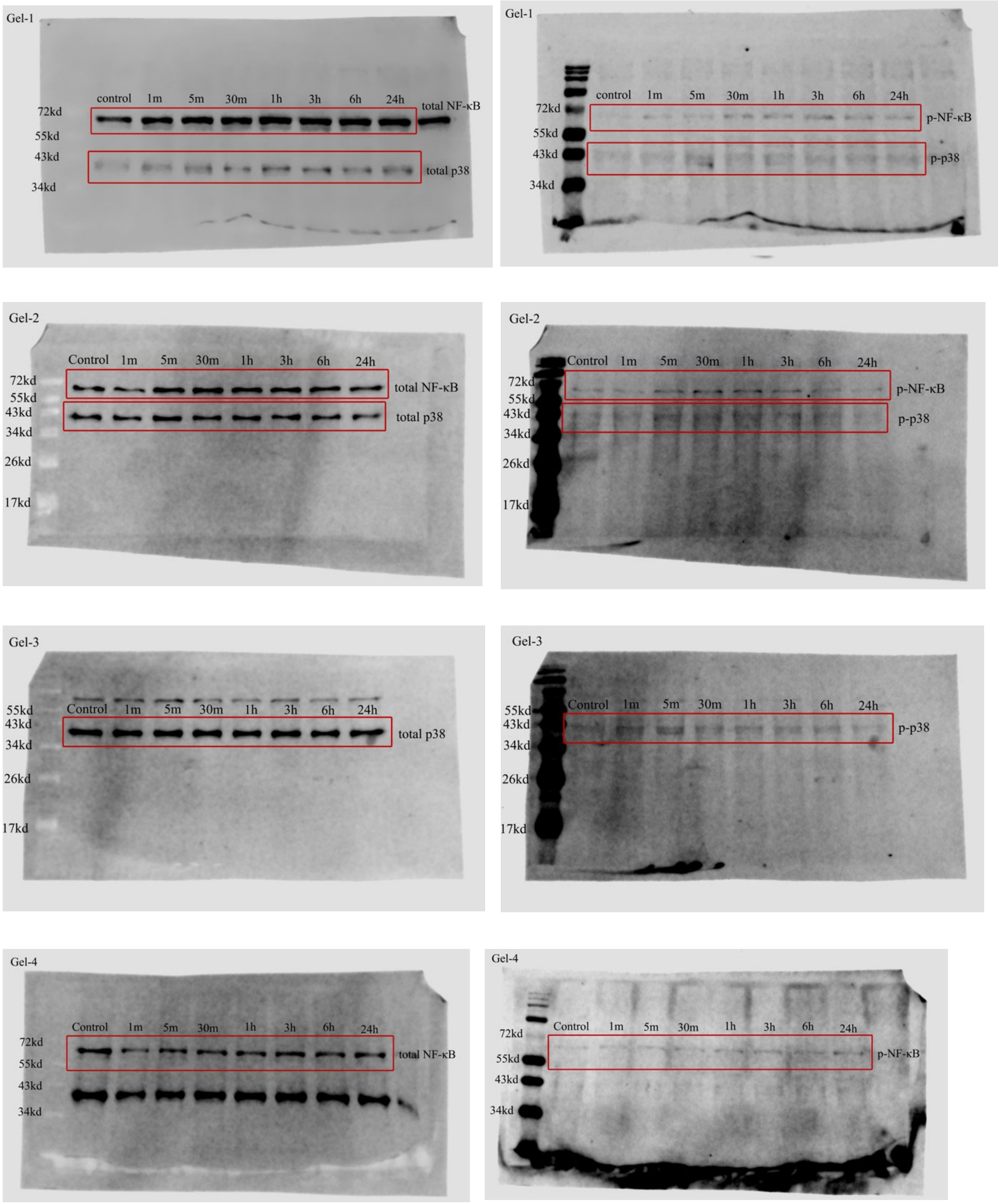

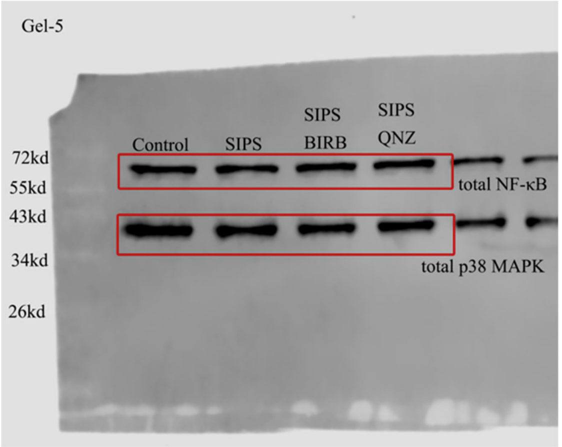
Gel-5
Control
SIPS
SIPS BIRB
SIPS QNZ
72kd
55kd
43kd
34kd
26kd
total NF-κB
total p38 MAPK

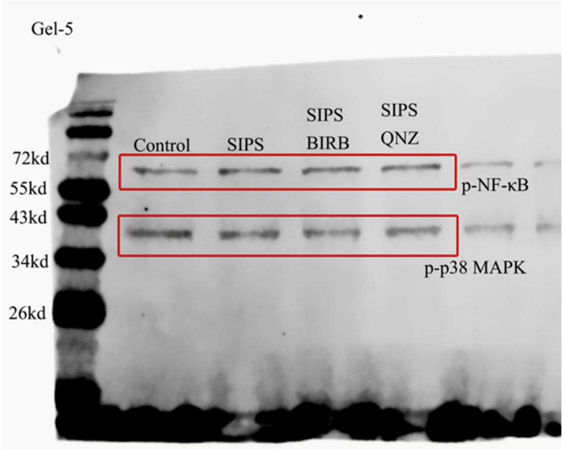
Gel-5
Control
SIPS
SIPS BIRB
SIPS QNZ
72kd
55kd
43kd
34kd
26kd
p-NF-κB
p-p38 MAPK

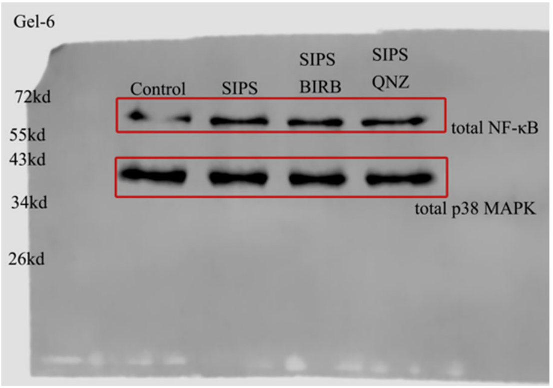
Gel-6
Control
SIPS
SIPS BIRB
SIPS QNZ
72kd
55kd
43kd
34kd
26kd
total NF-κB
total p38 MAPK

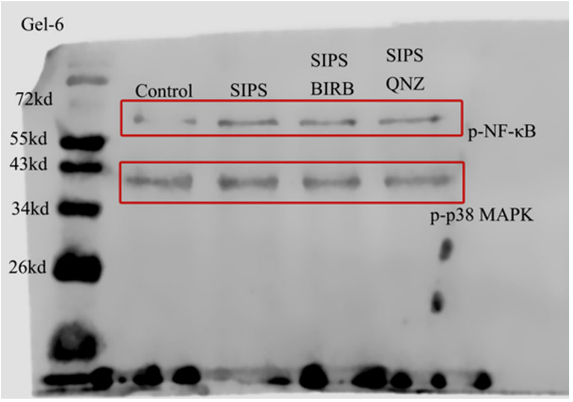
Gel-6
Control
SIPS
SIPS BIRB
SIPS QNZ
72kd
55kd
43kd
34kd
26kd
p-NF-κB
p-p38 MAPK

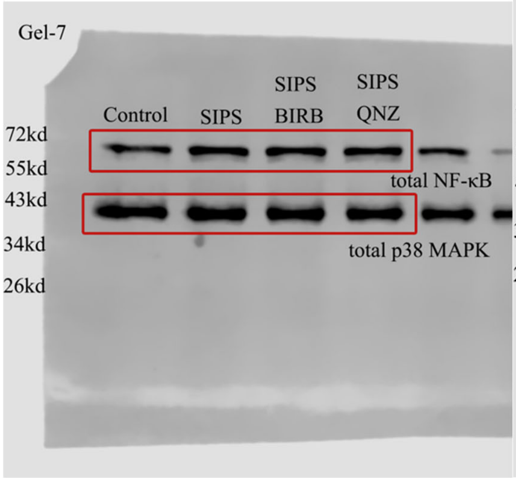
Gel-7
Control
SIPS
SIPS BIRB
SIPS QNZ
72kd
55kd
43kd
34kd
26kd
total NF-κB
total p38 MAPK

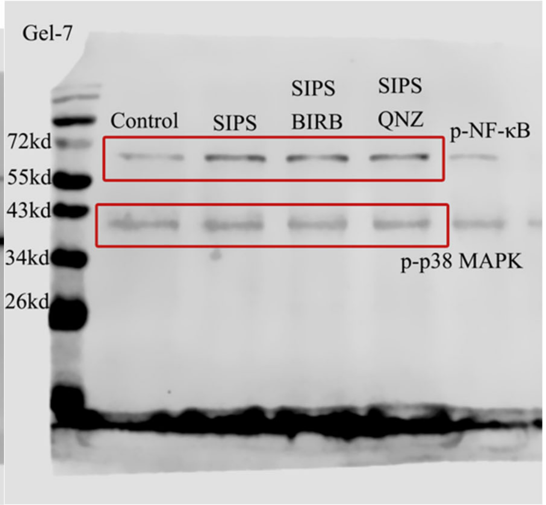
Gel-7
Control
SIPS
SIPS BIRB
SIPS QNZ
72kd
55kd
43kd
34kd
26kd
p-NF-κB
p-p38 MAPK

Figure S3. Original western blots and zymography images for figure 7. Bands on the membranes were captured using two different channels simultaneously i.e. 800 nm (anti-rabbit secondary antibody) and 680 nm channels (anti-mouse secondary antibody). Due to high molecular weight of ZO-1, the same protein samples were run on a separate gel to detect β-actin.

<u>ZO-1</u>

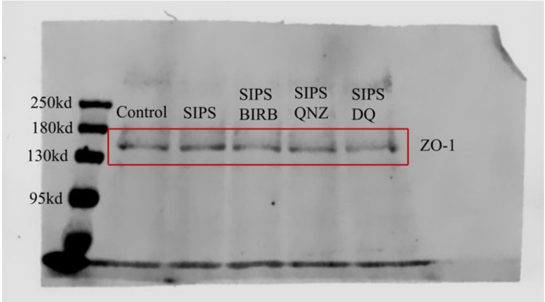

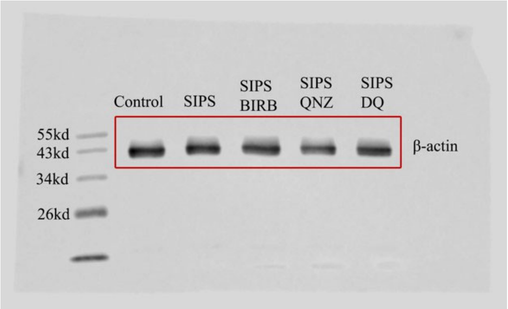

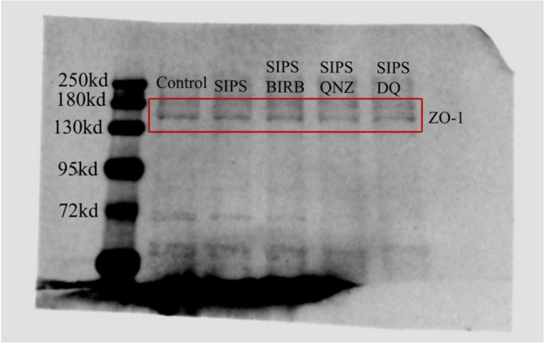

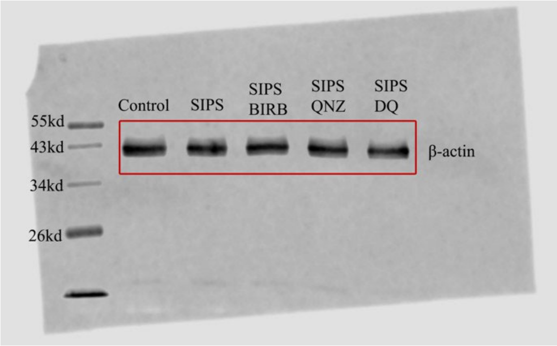

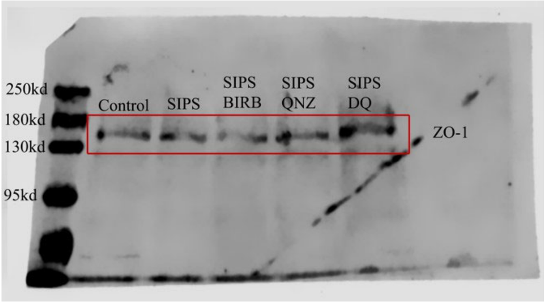

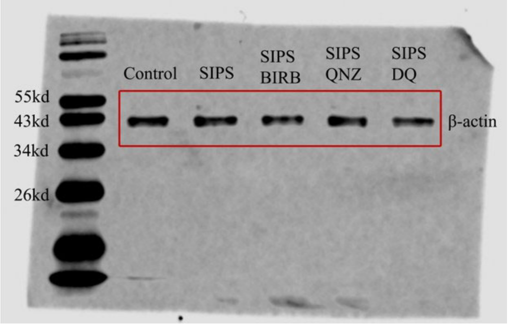

<u>Claudin-5</u>

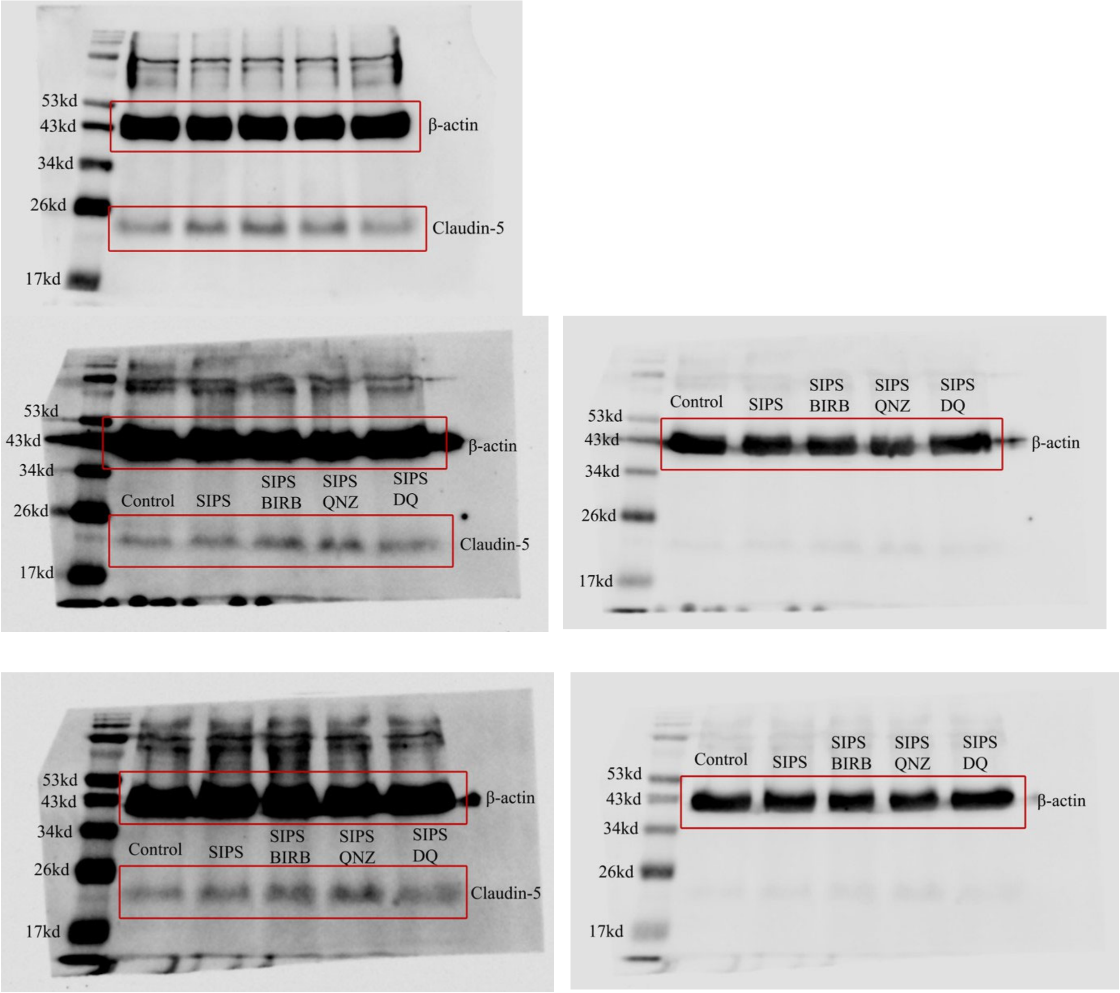

<u>Occludin</u>

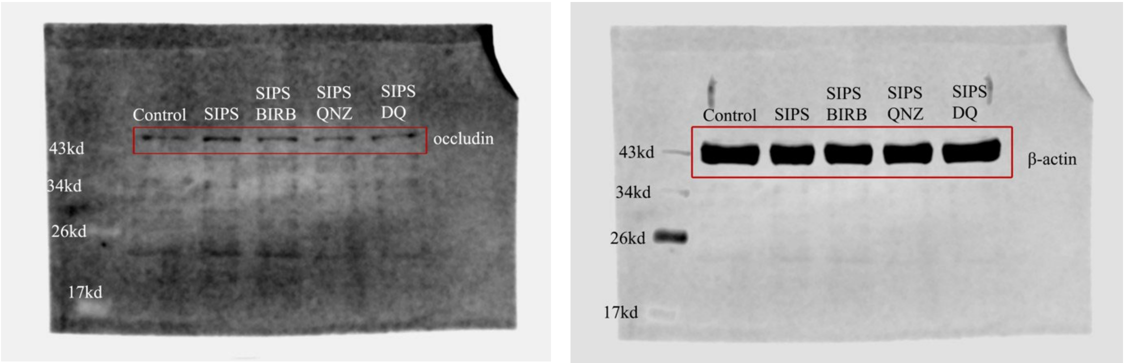

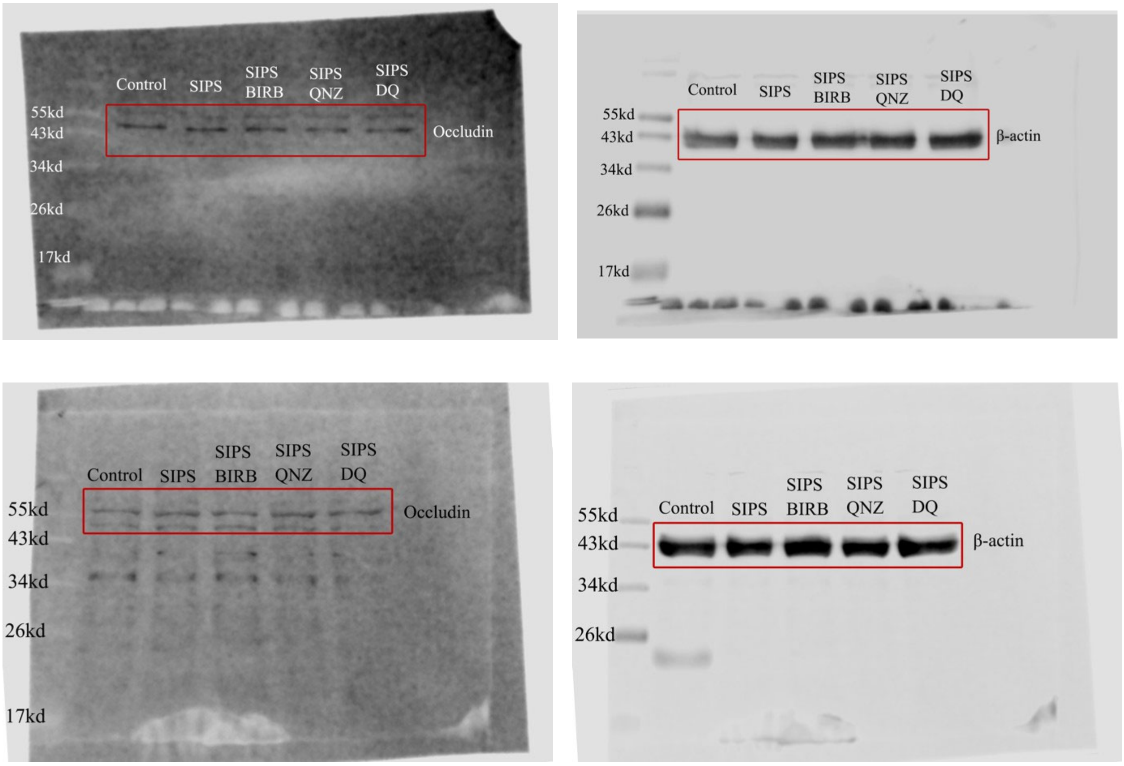
Control
SIPS
SIPS BIRB
SIPS QNZ
SIPS DQ
55kd
43kd
34kd
26kd
17kd
Occludin
β-actin

Zymography

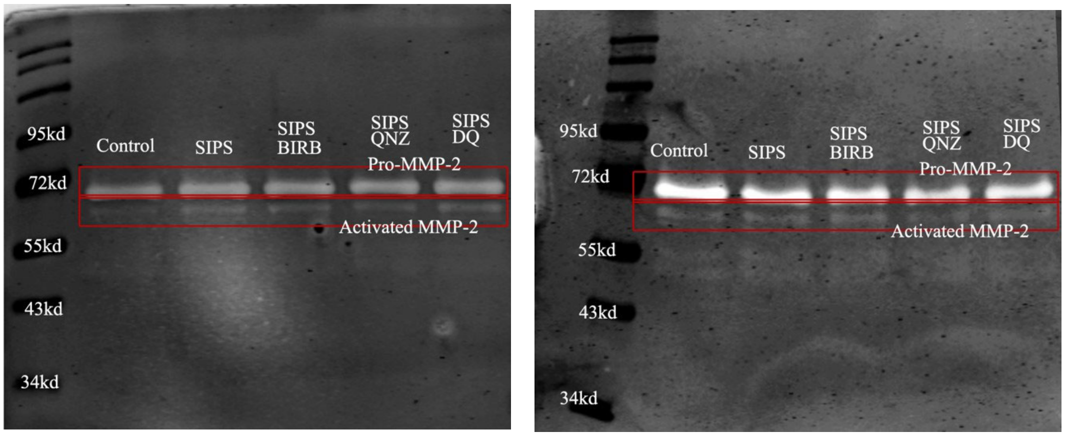
95kd
72kd
55kd
43kd
34kd
Control
SIPS
SIPS BIRB
SIPS QNZ
SIPS DQ
Pro-MMP-2
Activated MMP-2

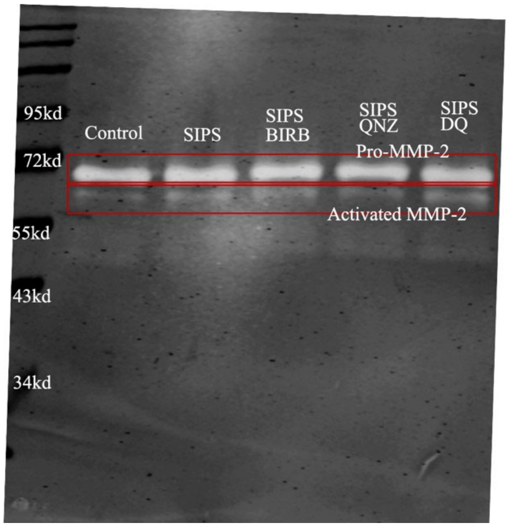
95kd
72kd
55kd
43kd
34kd
Control
SIPS
SIPS BIRB
SIPS QNZ
SIPS DQ
Pro-MMP-2
Activated MMP-2

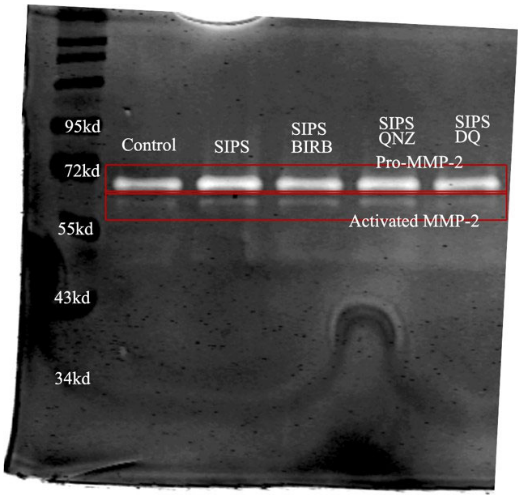
95kd
72kd
55kd
43kd
34kd
Control
SIPS
SIPS BIRB
SIPS QNZ
SIPS DQ
Pro-MMP-2
Activated MMP-2

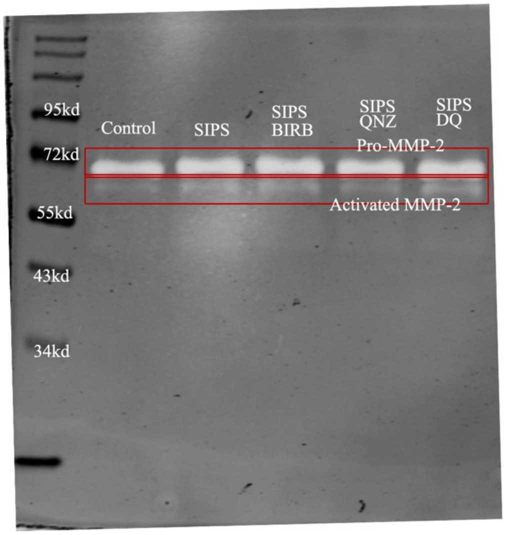
95kd
72kd
55kd
43kd
34kd
Control
SIPS
SIPS BIRB
SIPS QNZ
SIPS DQ
Pro-MMP-2
Activated MMP-2
